# Chromosome-Scale Assembly of Winter Oilseed Rape *Brassica napus*

**DOI:** 10.3389/fpls.2020.00496

**Published:** 2020-04-28

**Authors:** HueyTyng Lee, Harmeet Singh Chawla, Christian Obermeier, Felix Dreyer, Amine Abbadi, Rod Snowdon

**Affiliations:** ^1^Department of Plant Breeding, Justus Liebig University Giessen, Giessen, Germany; ^2^NPZ Innovation GmbH, Holtsee, Germany

**Keywords:** winter oilseed rape, genome assembly, long reads, *Brassica napus*, crop genomics

## Abstract

Rapeseed (*Brassica napus*), the second most important oilseed crop globally, originated from an interspecific hybridization between *B. rapa* and *B. oleracea*. After this genome collision, *B. napus* underwent extensive genome restructuring, via homoeologous chromosome exchanges, resulting in widespread segmental deletions and duplications. Illicit pairing among genetically similar homoeologous chromosomes during meiosis is common in recent allopolyploids like *B. napus*, and post-polyploidization restructuring compounds the difficulties of assembling a complex polyploid plant genome. Specifically, genomic rearrangements between highly similar chromosomes are challenging to detect due to the limitation of sequencing read length and ambiguous alignment of reads. Recent advances in long read sequencing technologies provide promising new opportunities to unravel the genome complexities of *B. napus* by encompassing breakpoints of genomic rearrangements with high specificity. Moreover, recent evidence revealed ongoing genomic exchanges in natural *B. napus*, highlighting the need for multiple reference genomes to capture structural variants between accessions. Here we report the first long-read genome assembly of a winter *B. napus* cultivar. We sequenced the German winter oilseed rape accession ‘Express 617’ using 54.5x of long reads. Short reads, linked reads, optical map data and high-density genetic maps were used to further correct and scaffold the assembly to form pseudochromosomes. The assembled Express 617 genome provides another valuable resource for *Brassica* genomics in understanding the genetic consequences of polyploidization, crop domestication, and breeding of recently-formed crop species.

## Introduction

*Brassica napus* subsp. *oleifera*, commonly known as rapeseed or canola, is the second most important oilseed crop globally (Food and Agriculture Organization of the United Nations, 2019). It originated from a natural hybridization event between *B. rapa* (AA, 2n = 2x = 20) and *B. oleracea* (CC, 2n = 2x = 18) no more than 7.5 1000 years ago (Chalhoub et al., 2014). Rapeseed was already widely cultivated in Europe from the 15^th^ to 18^th^ centuries for lamp fuel and soap production (Appelqvist and Ohlson, 1972). Following the introduction of double-low varieties (with low erucic acid and low glucosinolates in the seed) in the 1970s, modern rapeseed/canola varieties today deliver a high-value vegetable oil which can also be used for biodiesel production, while the extraction meal provides a high quality, protein-rich animal feed (Friedt and Snowdon, 2009). Oilseed rape is also a major component of crop rotations in most cereal-dominated agricultural systems (Friedt et al., 2018).

*Brassica napus* has an allotetraploid genome composition (AACC, 2n = 4x = 38) (Nagaharu, 1935; Allender and King, 2010). The formation of an allotetraploid involves the challenge to combine subgenomes of distinct species, with individual evolutionary history and epigenetic patterns, into one (Osborn et al., 2003; Comai, 2005). Studies of synthetic allopolyploids and natural neo-allopolyploids showed that the hybridization process of divergent genomes causes instant and prolonged alteration of gene expression, DNA methylation patterns and transposable elements regulation (Salmon et al., 2005; Lukens et al., 2006; Buggs et al., 2010; Chelaifa et al., 2010; Coate et al., 2014; Rigal et al., 2016; Edger et al., 2017). The genomic sequences of allopolyploids are also restructured as a result of illicit pairing of non-homologous chromosomes during meiosis, which encourages homoeologous exchange (HE) events (Gaeta et al., 2007; Xiong et al., 2011). HEs result in the replacement of chromosomal segments of one subgenome with another, and is hypothesized to lead to genome diploidization through fixation of HEs with time (Lysak et al., 2007; Mandáková et al., 2010). In *B. napus*, HEs were revealed through extensive structural rearrangements when the genome of a natural line was compared to the ancestral progenitors (Chalhoub et al., 2014). When compared among seven diverse *B. napus* genotypes, both shared and specific HEs up to a few 100 kb in size were found (Chalhoub et al., 2014), suggesting that HE is an ongoing process in *B. napus*. The mixture of older, fixed HEs and newly-formed HEs explains the wide-spread variations, such as reciprocal (Lombard and Delourme, 2001; Osborn et al., 2003; Piquemal et al., 2005) and non-reciprocal (Udall et al., 2005) translocations, between genotypes. These genotype-specific HEs have been shown to give rise to novel genetic diversities related to important agronomic traits such as flowering time (Pires et al., 2004; Chalhoub et al., 2014; Schiessl et al., 2017), leaf morphology (Osborn et al., 2003; Gaeta et al., 2007), and seed content (Harper et al., 2012; Qian et al., 2016).

The motivation of producing a highly-contiguous *B. napus* genome is clear, particularly from the aspects of breeding research. Genomes of high contiguity enable accurate design of SNP markers to obtain uniquely-mapped probes for marker-assisted selection. A direct comparison of GWAS and genomic selection results between highly-fragmented and chromosomal-scale assemblies of the blueberry genome shows better predictive ability and narrowing of QTL regions (Benevenuto et al., 2019). Similarly, high-quality genomes of the bread wheat (The International Wheat Genome Sequencing Consortium (IWGSC) et al., 2018) and its progenitors (Avni et al., 2017; Luo et al., 2017; Zhao et al., 2017; Ling et al., 2018) have enabled novel gene-to-trait discoveries such as dissection of shattering (Avni et al., 2017) and powdery mildew resistance (Ling et al., 2018). A complete genome assembly also helps fine-tune various decisions in breeding programs, such as target positions for genomic introgressions and identification of potential targets for genome editing CRISPR-Cas9 technologies (Gao, 2018). The genomic characteristics of *B. napus* increase the complexity of studying the genome sequences in three major respects. Firstly, homoeologous regions between subgenomes hamper the genome assembly process due to low sequence specificity. Ambiguities of highly similar sequences are difficult to resolve for assembly algorithms, particularly during the read clustering process (Nagarajan and Pop, 2013). In *B. napus*, reads originating from homoeologous regions cannot always be accurately assigned to individual subgenomes, and subgenomic distinction is further blurred by ongoing HE events. Recent assemblers adopt the *k*-mer binning method to resolve haplotypes using parental genome assemblies (Koren et al., 2018). Since high-quality assemblies of the *B. napus* progenitors are available (Belser et al., 2018), this approach could be plausible however it is nevertheless unable to fully resolve HE events, which interfere with subgenomic separation of *k*-mers. Secondly, as in many other complex crop genome assemblies, the high content of repetitive sequences in *B. napus* interfere with the construction of continuous chromosomes. The two diploid progenitors *B. rapa* and *B. oleracea* are both products of multiple paleopolyploidization events, where large-scale rearrangements occur following divergence from a common ancestor (Parkin et al., 2005). As a result, *B. napus* has potentially accumulated up to 72x multiplication since the origins of angiosperms and about 34.8% of the genome are estimated to be repeats (Chalhoub et al., 2014). Thirdly, the genome assembly of any single cultivar always fails to capture the entire genomic repertoire in a species, hence the need to use a pangenome as reference is recognized in crops (Tao et al., 2019). In oilseed rape, this need is highlighted by the HE-driven variations found between cultivars. Recent evidence shows ongoing HEs even in homozygous cultivars during self-pollination (He et al., 2017), suggesting that the variations between individuals of the same cultivar could be largely underestimated.

Genome assemblies for three cultivars of *B. napus* have been published to date (Chalhoub et al., 2014; Bayer et al., 2017; Sun et al., 2017), with Darmor*-bzh* and Tapidor being the two winter-type genotype represented. The Darmor*-bzh* genome is widely used as a standard reference genome from studies ranging from gene loss (e.g., Hurgobin et al., 2018) to SNP marker-assisted analyses like genome wide association studies (GWAS) (e.g., Gabur et al., 2018). However, all three genome assemblies were constructed prior to the advance of long-read technologies, therefore these assemblies are highly fragmented. To illustrate, the N50 read length of an Oxford Nanxopore MinION single flowcell run today is about 32 kbp (Supplementary Table S2), approximately the same size as the N50 contig length in the Darmor*-bzh* assembly (Chalhoub et al., 2014). The long-read technologies, led today by Pacific Biosciences (Eid et al., 2009) and Oxford Nanopore Technologies (Loman et al., 2015), revolutionized genomic research by producing continuous sequences of 10s to 100s of kilobases in length. They are now used to resolve complex and repetitive regions in plant genomes (for example Schmidt et al., 2017; Belser et al., 2018). Long-reads are therefore well-suited to resolve the aforementioned complications in assembling the *B. napus* genome by encompassing HE breakpoints and transposable elements.

Here we report the sequencing and genome assembly of the German winter oilseed rape accession ‘Express 617’ using 54.5x coverage with Pacific Biosciences long reads. Express 617 is a natural winter oilseed rape accession widely used in many existing mapping populations for linkage analyses of traits such as seed quality (Badani et al., 2006; Stein et al., 2013, 2017), seed yield and yield architecture (Radoev et al., 2008), heterosis (Basunanda et al., 2010) and disease resistance (Obermeier et al., 2013). Short reads, optical map data and genetic maps were used to further correct and scaffold the assembly to form pseudochromosomes. The Express 617 genome is assembled to 925 Mb in size, approximate to the flow cytometry estimation of 1132 Mb (Johnston et al., 2005). The base accuracy and pseudomolecule contiguity were validated using short read libraries, SNP markers and long read alignments. The genome was annotated to contain 12.5% of coding sequences (89857 predicted genes) and 37.5% of repetitive elements. The assembly was also compared to two other published *B. napus* genomes to identify collinear regions. A total of 56 same-chromosome collinear blocks of 488 Mb in size were identified in Express 617 (53%) when compared to the Darmor-*bzh* genome. In comparison, only 230 Mb (25%) of Express 617 are collinear with the ZS11 genome. This long-read genome of *B. napus* is expected to contribute to further understanding of HE in *B. napus* and its role in generation of genetic diversity for quantitative trait expression (Gabur et al., 2019). This assembly expands the genomic repository of *B. napus*, particularly for winter-type accessions, and consequently promotes exploitation of genomics advancement in oilseed rape and canola breeding programs.

## Results

### The Express 617 Genome Assembly, Gene Set, and Repetitive Elements

The total size of Express 617 genome assembly is 925 Mb, where placed pseudochromosomes are 765 and 160 Mb remained as unplaced random scaffolds (Supplementary Table S3). As shown in Table 1, this genome size is larger than three previously published assemblies, whereas the percentage of N-bases (quantity of gaps) is lowest among all five assemblies. The high contiguity of Express 617 is also reflected in the length of N50 scaffolds (4.8 Mb) prior to pseudochromosome construction.

**TABLE 1:** Assembly statistics of the Express 617 genome in comparison with three previously published *B. napus* genome assemblies.

	Darmor-*bzh* v4.1 (Chalhoub et al., 2014)	Darmor-*bzh* v8.1 (Bayer et al., 2017)	ZS11 (Sun et al., 2017)	Tapidor (Bayer et al., 2017)	Express 617
Total genome size (Mb)/percentage of Ns	850/13.17%	850/13.16%	976/7.05%	636/5.16%	925/0.09%
Length of pseudochromosomes (Mb)	645	798	854	627	765
Length of unplaced scaffolds (Mb)	204	51	120	8.4	160
Number of scaffolds prior to pseudochromosome construction	20702	-	3460	21280	1632
Length of N50 scaffold prior to pseudochromosome construction (bases)	763688	-	602220	197031	4882293

The genome consists of 12.5% coding sequences, 89857 genes with 99481 transcripts (Table 2) and 37.5% repetitive elements (Supplementary Table S4). The transcripts have an average length of 1924.8 bp, with an average of 5.22 exons each. Average lengths of intron and exon are 183.3 and 226.5 bp, respectively. A total of 87951 transcripts contain at least one known protein domain that can be found in curated protein databases. As observed in all other plant genomes, the majority of the Express 617 repeats are long terminal repeats (LTRs) (28.3% of all repetitive bases masked), where 22.2% are Gypsy and 16.8% Copia retrotransposons. The non-LTR subclass I and subclass II comprise 4.9 and 13.5%, respectively, while the remaining transposable elements remain uncharacterized (25.5%). Satellites, simple repeats and low complexity sequences make up another 5.2% of all repeat sequences.

**TABLE 2:** Gene annotation and evaluation of the Express 617 genome.

	Express 617
Number of genes	89857
Number of transcripts	99481
coreGF	Weighted score: 0.95
	Number of missing coreGFs: 159
Number of BUSCOs found	Total complete: 4358 (94.8%)
	Complete single copy: 866
	Complete duplicated: 3492
	Fragmented: 11
	Missing: 227
Number of transcripts aligned to pan-transcriptome	87012
Number of proteins containing InterPro domains	87951

Consistent with previous studies (Chalhoub et al., 2014), the chromosomes of subgenome A have higher gene density with lesser repetitive elements when compared to subgenome C (Supplementary Figure S1). This is explained by subgenomic dominance, a phenomenon documented in many polyploids such as cotton (Renny-Byfield et al., 2015) and maize (Schnable et al., 2011), where homoeologous copies of “dispensable” genes are preferentially silenced (Edger et al., 2018).

### Evaluation of the Assembly Quality

We took multiple steps to avoid common errors in the assembly process, and then extensively evaluated the results. The correctness of the assembly was evaluated in three ways, (1) base-level errors, (2) large-scale translocations, and (3) completeness of the gene set.

Base-level errors are single nucleotide mutations and short indels that usually arise from the sequencing process. The error rate of raw PacBio long reads was estimated to be up to 15% (Korlach, 2013). Using the alignment of two libraries of Illumina short reads, we assessed the error rate of a subset of PacBio reads (10% of total nucleotides in all reads). By allowing single-end mapping, 7% of the total nucleotides of mapped reads were mismatches, which is half of the maximum estimated error assuming that Illumina reads have near-to-zero sequencing errors (Glenn, 2011). To reduce the effect of long read sequencing errors, we used consensus long reads that were generated by self-alignment. We also incorporated high coverage of short reads during the assembly, as well as post-assembly error correction. To measure the base-level accuracy of the genome, five libraries of Illumina sequencing reads, of which four were used to construct the assembly and one was sequenced independently, were used. A total of 89% of paired-end reads aligned concordantly in the correct direction and insert size, with zero mismatches and gaps (Supplementary Table S5).

Large structural error is a primary concern when assembling polyploidy genomes, particularly allopolyploids with frequent HEs like *B. napus* (Samans et al., 2017). These errors could manifest through a few assembly processes, for example (1) wrongly-placed scaffolds during the construction of pseudochromosomes due to non-specific matching to genetic maps, (2) short mate-paired libraries and linked-reads could be unspecific to differentiate between regions of homeoelogous chromosomes, and form wrongly-joined scaffolds, and (3) regions with high density of repetitive elements may form small scaffolds and could be wrongly-placed as described in (1). To evaluate large-scale errors, a combination of SNP markers and long read alignments was used. First, the distribution pattern of gene allelic SNPs in the AC *Brassica* genomics platform (He et al., 2015) generated by the genome-ordered graphical genotypes (GOGGs) method (He and Bancroft, 2018) was manually inspected. The correctness of the assembly was measured by low amount of alternating parental alleles in individual recombinant lines, with the assumption that allelic SNPs segregate across a mapping population while interhomeolog and interparalog SNPs do not. We detect a total of 24 regions of discording allelic patterns indicating putatively incorrect gene order, which could originate from incorrectly placed scaffolds or misjoin of scaffolds (example in Supplementary Figure S2). They were labeled as potentially misassembled. To confirm that they were indeed true misjoins and not inaccuracies introduced during GOGG such as ambiguously-mapped orthologs, we used alignments of long reads to the assembly. Long read alignments were generated using PacBio reads which were used to construct the assembly and additionally 17x of Nanopore ONT reads. A total of 86% (562142) of the Nanopore reads and 99% (4328786) of PacBio reads aligned to the assembly. True misjoins were identified by refining the resolution of breakpoints, which are characterized as a huge decrease of mapped reads and an enrichment of split-reads, as seen in Supplementary Figure S3. When supported by high coverage of mapped reads for both PacBio and Nanopore technologies, a putatively misassembled region was dismissed, as shown in Supplementary Figure S4. Using read coverage as supporting evidence, a total of seven regions, ranging from 123 kb to 3 Mb were identified as misassemblies. All cases of true errors have one or both breakpoints in stretches of Ns. Ns were introduced as gap-fillers during the construction of pseudochromosomes and scaffolding of using linked-reads. Regions with high frequency of Ns therefore symbolizes difficult regions where their local sequence proximity, termed “edges” in an assembly graph (Wick et al., 2015), cannot be resolved. These regions were extracted and retained as unplaced scaffolds. Supplementary Figure S5 shows the final arrangements of scaffolds based on the genetic versus physical distance of a total of 24469 markers (17478 in Express 617 × R53; 8469 in Express 617 × V8; 12140 in Express 617 × SGDH14) in each pseudochromosome. The pseudochromosomes were also compared to the progenitors *B. rapa* and *B. oleracea* genomes to show that sequence similarities of subgenomes are as expected (Supplementary Figure S6).

The completeness of predicted genes were evaluated with a set of well-conserved genes across plant species using PLAZA coreGF (van Bel et al., 2012) and BUSCO (Simão et al., 2015). Out of 2928 core green plants gene families in PLAZA, 2803 were identified in the predicted gene set, therefore obtaining a weighted score of 0.95. BUSCO (v4.0.4) detected 4358 (94.8%) out of 4596 complete orthologous groups within the Brassicales lineage dataset, with 3492 being duplicated and 866 being single copy. In comparison (Supplementary Figure S7), version 4.1 of Darmor*-bzh* has 4378 (95.2%) complete BUSCOs, version 8.1 of Darmor*-bzh* has 4379 (95.2%), ZS11 has 4263 (92.7%) and Tapidor has 4162 (90.6%). Additionally, a publicly-available single-ended RNAseq library was used to ballpark the accuracy of annotated introns. A total of 342674 introns were predicted using RNAseq, where 229278 (67%) matched to the introns annotated, 104883 overlapped with predicted gene region, and 8513 were in intergenic regions. This indicates that 62% of introns annotated are supported by external data, which was not used in the annotation pipeline.

### Comparison Between Express 617 and Other *B. napus* Genomes

**FIGURE 1:**
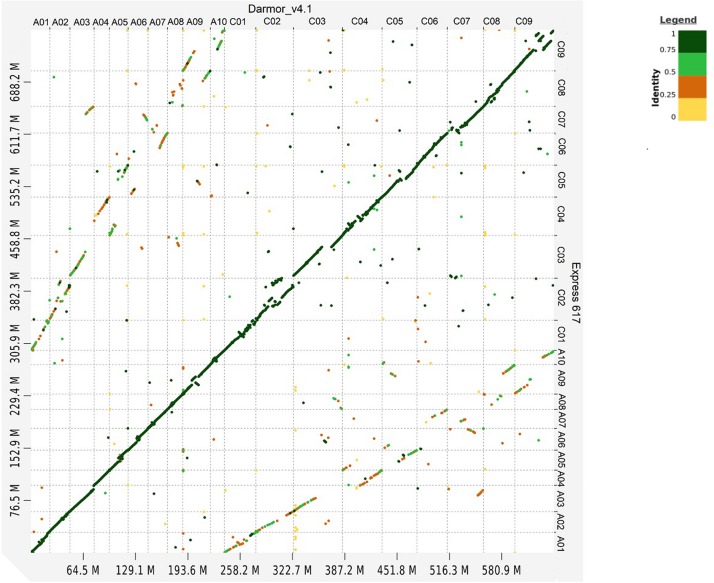
Dot plot comparison between Express 617 and Darmor-*bzh* v4.1 genome. Sequence similarity is color coded from 0 to 1.

Whole genome alignment between Express 617 and Darmor-*bzh* shows high sequence similarity in all chromosomes (Figure 1). The secondary alignments of lower similarity between homeologous chromosomes can also be observed.

**FIGURE 2:**
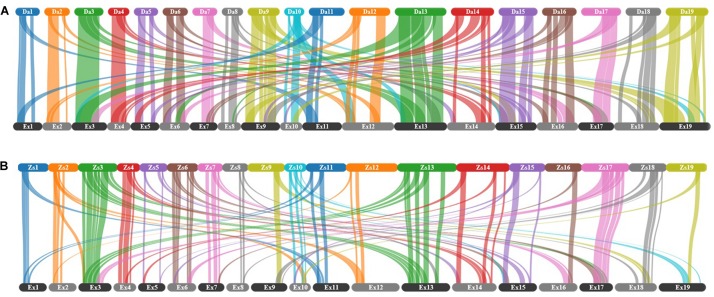
Collinearity between Express 617 and two other *B. napus* assemblies for all chromosomes. **(A)** Darmor*-bzh* v4.1 versus Express 617, **(B)** ZS11 versus Express 617. Collinear blocks are indicated as connecting bars between genomes. The chromosomes were labeled with two letter indicating the cultivar followed by chromosome number, where 1 to 10 corresponds to chromosomes A1 to A10 and 11 to 19 corresponds to chromosomes C1 to C9.

To examine the shuffling of chromosomal segments, the gene-level collinearity between genomes, which is defined as the conservation of gene order within syntenic regions (Coghlan et al., 2005), was identified. A total of 120 collinear blocks, linked by 77840 gene pairs, were found between Express 617 and Darmor-*bzh* (Figure 2A). Out of 120, 56 blocks linked by 45410 gene pairs correspond to the same chromosomes. These 56 blocks made up 488 Mb (53%) of the Express 617 genome and 425 Mb (57%) of the Darmor*-bzh* genome. In comparison, Express 617 shared 100 collinear blocks (38145 gene pairs) with ZS11 (Figure 2B), a spring oilseed rape line, where 56 of the blocks (21982 gene pairs; 230 Mb of Express 617 and 274 Mb in ZS11) are in the same chromosomes.

## Discussion

Improved contiguity of Express 617 genome in comparison to other *B. napus* assemblies is evident in the low number of scaffolds, high N50 scaffold length and the low percentage of Ns in total genome size (Table 1). This improvement is expected as the all other four are short read assemblies. The Tapidor assembly has the lowest contiguity as it was assembled with about 30x of Illumina short reads and the contigs were placed using SNPs (Bayer et al., 2017). ZS11 and Darmor-*bzh* were constructed with more comprehensive data, including higher coverage of short read sequencing, long range mate-paired reads and BAC-by-BAC approach (Sanger-sequenced for Darmor-*bzh* and Illumina-sequenced for ZS11) (Chalhoub et al., 2014; Sun et al., 2017). For both assemblies the general approach was that first BAC sequences were used to form contigs, gaps were filled with short reads, and then genetic maps were used to place contigs. Since the maximum read length was 100 bp, the assemblies produced are enriched with gaps, as reflected on the percentage of Ns. Better contiguity also means that the intergenic and repeat-rich regions such as centromere are better assembled. This will enhance the development in molecular breeding such as application of transposable element markers (Bhat et al., 2020), following increasing understanding of the role of transposable elements in crops, such as in disease resistance [example in pepper (Kim et al., 2017)], domestication [example in rice (Li et al., 2017)], and adaptations [example in maize (Lai et al., 2017)].

A considerable amount of scaffolds (160 Mb) cannot be placed in the Express 617 pseudochromosomes. Due to the subgenomic similarities and frequency of HEs, a relatively conservative approach was taken to construct this assembly. To avoid false positives and wrong conclusions led by misassemblies, the assembled scaffolds were broken in two independent steps, therefore trading off contiguity for accuracy. Misjoins were broken first during the incorporation of optical map data, and second during GOGG evaluation. Optical maps provide independent long-range evidence for the connection of scaffolds. However, in the circumstances of conflicts between optical map and the assembled sequences, a decision has to be made to resolve conflicts. Since optical maps are not error-free (Jiao et al., 2017), the software *Chimericognizer* (Pan and Lonardi, 2019) used alignments adjacent to the conflicts to estimate the confidence of chimeric sites. A total of 92 scaffolds (out of 1547) in the assembly were identified as chimeric and broken to form 206 scaffolds. Stitching of all the scaffolds was then attempted next using *Novo&Stitch* (Pan et al., 2018). Similarly, using the GOGG approach followed by long read mapping, a total of seven regions were identified as misjoins and breakpoints were cut. Even though the correct chromosome and position of these misassembled blocks can be identified using GOGG patterns, they are of low resolution. In other words, there is no way to accurately determine a breakpoint for insertion of these blocks. These blocks were therefore retained as random scaffolds, with the putative chromosome appended to the scaffold name (Supplementary Table S3). Out of all unplaced scaffolds, 38 Mb were assigned to chromosomes and contain 2946 genes, whereas 122 Mb contain 2803 genes, with 34.3% of them being repetitive elements.

The completeness of gene space is one of the ways to evaluate an assembly (Veeckman et al., 2016). Coding sequences made up of 12.5% in Express 617, comparable to 11.9% in Darmor-*bzh* v4.1 (Chalhoub et al., 2014). Both coreGF and BUSCO indicate a 95% completeness of conserved orthologous groups in Express 617 genome. Based only on BUSCO results, this is comparable to Darmor-*bzh*, where 20 more orthologous groups were identified, and more superior to Tapidor and ZS11 (Supplementary Figure S7). We postulated that the error rate of long read sequencing affect the accuracy of gene prediction, as observed in human genome assemblies (Watson and Warr, 2019). This could possibly also be reflected on the lower number of confident genes in Express 617 when compared to Darmor-*bzh.* Using Illumina short reads as a benchmark, PacBio raw reads have an overall mapping rate of 71% and an error rate of 7%, whereas the assembly has an overall mapping rate of 99% (perfect mapping rate of 89%) and an error rate of 0.5%. This improvement is largely contributed by the pre-assembly consensus read construction and multiple rounds of short read polishing. We anticipate better polishing softwares, such as sequencing signal-based tools (such as Nanopolish^1^) to resolve the 0.5% uncorrected errors. Nevertheless, we argue that from the perspective of the amount of resources and time used, long read technology has definitely increased the efficiency to produce high quality genomes. For example, the BAC-by-BAC pooled strategy used in Darmor-*bzh* is known to be highly accurate yet expensive and include labor-intensive processes such as fingerprinting clones.

Another possibility for undetected errors to persist in this assembly is that when correlating genetic maps to physical positions, two assumptions were used (1) the genetic maps accurately represent the Express 617 genome, and (2) each marker probe mapped correctly to the chromosomal position of origin. However, even though Express 617 is the common parent of three populations used to generate genetic maps used, there are two populations with parents of synthetic backgrounds (R53 and V8). Synthetic accessions are known to contain more HEs than non-synthetics (Sharpe et al., 1995; Liu et al., 2014; Rousseau-Gueutin et al., 2017; Hurgobin et al., 2018). For example, a large part of chromosome C02 in R53 was known to be replaced by A02 (Stein et al., 2017). To reduce the manifestations of these HEs in Express 617 pseudochromosomes, weighted priority was given to population of natural lines (Express 617 × SGDH14). The limitation of the second assumption is the specificity of probe mapping. As the length of marker probe is only 50 bp, it could map to multiple positions (8227 out of 44113 of mapped probes are non-unique). Even with uniquely mapped probes, scaffolds could still be wrongly assigned to homeologous or similar regions of non-homeologous chromosomes. Homeologous mappings can be observed in Supplementary Figure S5, particularly between A01 and C01, A03 and C03, A09 and C08, and A09 and C08, which are known hotspots for HE events (Chalhoub et al., 2014; Lloyd et al., 2018). Also, the density of markers are not consistent along the chromosomes. To illustrate, the first misjoin in chromosome A01 (position 1846993) detected by GOGG only have adjacent markers at a 58 kbp distance upstream and 139 kb downstream. Since only uniquely-mapped markers were used, repetitive or highly homoeologous regions contribute to large gaps between markers. This potentially explains how the misjoin was formed during the assembly, and how it was not detected with 10x linked reads and optical mapping.

*Brassica napus* morphotypes cluster into winter, semi-winter and spring growth habits based on SNPs (Diers and Osborn, 1994; Becker et al., 1995; Bus et al., 2011; Gazave et al., 2016; Delourme et al., 2018) and show sequence and copy number variation in flowering time regulatory genes (Schiessl et al., 2017). Collinearity comparisons between the two winter-type cultivars Express 617 and Darmor-*bzh*, and between Express 617 and the Chinese semi-winter cultivar ZS11 reflected this expectation of genetic diversity. However, we nevertheless cannot disregard the influence of assembly quality and completeness in collinearity studies. Regions that are not collinear could arise from true genetic diversity, assembly artifacts such as misassemblies and gap regions, or unidentified genes. Repetitive elements in the genome are likely to be the major contributor of these regions. For example, repeat-masking approach was found to be the main cause of varying number of repeat-containing disease resistance genes in four *B. napus* genomes, instead of true biological variations (Bayer et al., 2018).

## Materials and Methods

### Whole Genome Sequencing of Plant Material

#### Illumina and Pacific Biotechnologies Sequencing

Approximately 40 g of fresh leaf tissue was collected from an advanced inbred line (>F_11_) of the winter type oilseed rape accession “Express 617.” DNA libraries of 350 bp, 450 bp, 2 kbp, 5 kbp, and 10 kbp were constructed and subjected to paired-end sequencing on the Illumina HiSeq 2000 platform. The 20-kb SMRTbell library was prepared using SMRTbell Template Prep Kit 1.0-SPv3, where the qualified high-molecular weight DNA were fragmented to approximately 20 kb, followed by damage repair, end repair and adapter ligation. Size selection was then performed using BluePippin^TM^ Size-Selection System (Sage Science, Beverly, MA, United States). The quality of purified library was checked using Qubit (Invitrogen) and Advanced Analytical Fragment Analyzer (AATI). The SMRTbell-Polymerase Complex was prepared using Sequel^TM^ Binding Kit 2.0 and sequenced on Sequel SMRT Cell 1M v2. A 6 h movie using the Sequel Sequencing kit 2.0. 10x Genomics libraries also constructed and sequenced on the Illumina platform to produce GemCode linked reads. All sequencing described above was outsourced to Novogene, Co., Ltd. (China). Raw reads obtained were deposited to the NCBI Short Read Archive (PRJNA587046). Sequencing depths obtained for each library are recorded in Supplementary Table S1.

#### DNA Isolation for Oxford Nanopore Sequencing

The DNA isolation was carried out in accordance to high molecular weight DNA isolation protocol as described by Mayjonade et al. (2016). Approximately, 5 g of fresh leaves were harvested from rapeseed plants at 4–6 leaf stage. This frozen leaf was immediately frozen using liquid nitrogen. This frozen leaf was subjected to mechanical grinding using a mortar and pestle. 4–5 ml of pre-heated lysis buffer [1% (w/v) PVP10, 50 nM EDTA, 1.25% (w/v) SDS, 1% (w/v) Na_2_S_2_O_5_, 5 mM C_4_H_10_O_2_S_2_, 100 mM TRIS pH 8, 500 nM NaCl, 1% (v/v) Triton X-100, 1% (w/v) PVP40] to the frozen leaf samples for disrupting the cell wall. This was followed by incubation of the lysate for 30 min at 37°C. In order to precipitates sodium dodecyl sulfate (SDS) and SDS-bound proteins, 0.3 volumes of 5 M Potassium Acetate was added to the lysate and spun at 8000 *g* for 12 min at 4°C. Clean DNA was then recovered by fishing out the DNA using magnetic beads.

#### Size Selection and Library Preparation for Oxford Nanopore Sequencing

1–3 μg of DNA was subjected to size selection using Circulomics short-read eliminator XL kit (Circulomics, Inc., Baltimore, MD, United States) according to the manufacturer’s instruction. The kit uses selective precipitation to deplete DNA fragments shorter than 40 kb. The size selected DNA was then used for the preparation of the sequencing library, using SQK-LSK109 (Oxford Nanopore Technologies) kit in accordance with the manufacturer’s recommendations. Following the library preparation, DNA was finally loaded onto an Oxford Nanopore MinION flow cell (version R9.4.1) for sequencing. The raw fast5 files produced by the MinION device were then base-called using *Guppy* 3.0.3 (Oxford Nanopore Technologies) with “dna_r9.4.1_450bps_hac.cfg” model using standard parameters to generate fastq file. Supplementary Table S2 shows the statistics of reads generated.

### Optical Map Construction

DNA isolation for optical mapping was performed according to the IrysPrep^TM^ Plant Tissue-Nuclei protocol provided by BioNano Genomics. Nearly 2 grams of young leaves were harvested from dark-treated rapeseed plants, immediately followed by fixing with 2% formaldehyde. In order to isolate the intact nuclei, fixed leaf material was subjected to homogenization in an isolation buffer containing BME, Triton X-100 and PVP-10. Purified nuclei were then embedded into an agarose gel matrix. Finally, the DNA was recovered by melting the agarose plugs using GELase^TM^ (Epicentre) treatment. Sequence specific nick labeling using Nt.BspQI (recognition site GCTCTTC) was performed on the isolated DNA using the IrysPrep^TM^ Labeling-NLRS protocol by BioNano Genomics. Finally these single DNA molecules were loaded onto an IrysChip for imaging on the BioNano Genomics Irys platform. The DNA molecules were imaged using the BioNano Irys System and were computationally translated into single-molecule optical maps. Single optical maps were then assembled into a consensus map with IrysSolve pipeline (v5134) provided by BioNano Genomics, and deposited as Supplementary file in NCBI BioProject PRJNA587046.

### Genetic Maps Construction

Genetic maps were constructed for the two biparental populations Express 617 × R53 (ExR53-DH) and Express 617 × V8 (ExV8-DH) using 60K Illumina Infinium Brassica SNP array, SSR and AFLP marker data obtained from 244 and 216 lines, respectively. SNP and SNaP marker data were filtered according to Gabur et al. (2018). SSR and AFLP marker data were taken from the genetic maps produced by Radoev et al. (2008) and Basunanda et al. (2010). Genetic maps were constructed using the software *MSTMap* (Wu et al., 2008) applying the Kosambi map function. The genetic linkage map for Express 617 × SGDH14 was produced using 60K Illumina Infinium Brassica SNP array marker data obtained from 139 lines using the software package *JoinMap* 4.1 (Stam, 1993; van Ooijen, 2011) applying the Kosambi map function (Behnke et al., 2018).

### Genome Assembly

To increase read accuracy, PacBio raw reads were self-aligned to generate consensus reads using *Daligner* v1.0 (Myers, 2014) using default parameters. Consensus reads were then assembled using *FALCON* (falcon-2017.11.02-16.04-py2.7) (Chin et al., 2016) to form unitigs. Unitigs were then further polished using the consensus algorithm Quiver (SMRT Link v5.0.1)^2^. Illumina short reads were used to correct small-scale errors using default parameters of *Pilon* (pilon-1.18.jar) (Walker et al., 2014). To increase contiguity, PacBio reads were used to further scaffold the unitigs (SSPACE-standard) (Boetzer et al., 2011). 10x Genomics data were first processed by trimming the first 16 bp barcode and subsequence 7 bp random primer sequence of the first mate of each pair, and then aligned to the scaffolds to form super-scaffolds using *fragScaff* (version 140324) (Adey et al., 2014). Assembly procedures described above were performed by Novogene, Co., Ltd.

The obtained super-scaffolds were corrected for large-scale chimeric regions originating from misassembly by comparing to an Express 617 BioNano optical map using *Chimericognizer* (Pan and Lonardi, 2019) where junctions of chimeric scaffolds were broken with the following parameters “-a 1.5 -b 1 -d 25 -e 50000 -h 50000 -r 80000”. Scaffolds were then corrected using *Novo&Stitch* (Pan et al., 2018) with the default strict parameters that are equivalent to “-a 3000 -b 0.1 -c 10000 -d 0.5 -e 0.9 -h 25 -r 0.2”.

The corrected scaffolds were then arranged into pseudochromosmes using three high-resolution SNP-based genetic maps, including Express 617 × SGDH14, Express 617 × V8 and Express 617 × R53. Weighted priority 3, 2 and 1 were given to the listed maps respectively based on the synthetic origin of parents. Pseudochromosome construction was completed using the software *ALLMAPS* (Tang et al., 2015) with the following parameters “python –m jcvi.assembly.allmaps path –mincount = 10 –links = 25”. Scaffolds that map to multiple linkage groups were identified as potentially chimeric, and the breakpoints were detected using “python –m jcvi.assembly.allmaps split” and “python –m jcvi.assembly.patch refine”. Corrected pseudochoromosomes were produced by repeating the *ALLMAPS* run with the broken scaffolds.

### Gene Annotation

Repetitive sequences were identified using *RepeatModeler* (Smit and Hubley, 2008), a repeat family identification and modeling package which performs two *de novo* repeat-finding programs *RECON* (Bao, 2002) and *RepeatScout* (Price et al., 2005). The repeats identified were soft-masked in the assembly using *RepeatMasker* vopen-4.0.7 (Smit et al., 2013).

The gene prediction pipeline *BRAKER2* (Hoff et al., 2019) was used to train an Express 617-specific gene model and then predict genic regions. *BRAKER2* executes the gene predictor *Augustus* (Hoff and Stanke, 2013, 2018) internally. First, the proteomes of two species *Arabidopsis thaliana* (Proteome ID UP000006548) and *B. napus* (Proteome ID UP000028999) were aligned to the genome using *Genome Threader* (Gremme et al., 2005), and provided as evidence for model training in *Augustus*. The trained parameters were then used, together protein homology hints, to accurately predict genes. Fragmented predictions and potential pseudogenes were further filtered with (1) *Augustus* script “python Augustus/scripts/getAnnoFastaFromJoingenes.py –s TRUE” and (2) high identity to all 341468 proteins in the *Brassica* genus (Taxon identifier 3705) in UniProt release 2019_08 (The UniProt Consortium, 2019), with an alignment coverage of 80% to both target and query, a percentage identity of 80% and above, and -evalue 10e-5 using *BLASTP* (Camacho et al., 2009).

### Evaluation of Base-Level Accuracy

To estimate the error rate of PacBio reads, two Illumina sequencing libraries (SRR10382360 and SRR10382369) were aligned to the assembly using *Bowtie2* version 2.2.6 (Langmead and Salzberg, 2012) with the following parameters “bowtie2 -I 200 -X 500 –end-to-end –no-discordant”. Error rate was estimated by calculating the ratio of mismatch bases (“mismatches”) to total bases of mapped reads (“bases mapped”) from the output of samtools stats version 1.9 (Li et al., 2009).

Five Illumina sequencing libraries (SRR10382360, SRR10382369, SRR10382370, SRR10382371, SRR1030294) were aligned to the assembly using *Bowtie2* version 2.2.6 (Langmead and Salzberg, 2012) with the following parameters “bowtie2 –I 200 –X 500 –end-to-end –no-mixed –no-discordant”. Read pairs which align perfectly were counted with the following command “cat file.sam | grep -v “^@” | cut -f1,6 | uniq -c | grep 150M | grep -vc “1 ””. The same five libraries were also aligned to a subset of PacBio corrected reads.

### Evaluation of Scaffolding Accuracy

Nanopore sequences (SRR10383383) were first filtered by q10 using *NanoFilt* (De Coster et al., 2018) and corrected to replace sequencing noise with consensus using *Canu* (Schmidt et al., 2017) with the following parameters “-genomeSize = 1g -correctedErrorRate = 0.105 -minReadLength = 3000 -minOverlapLength = 2000 -corOutCoverage = 200 “batOptions = -dg 3 -db 3 -dr 1 -ca 500 -cp 50” -ovlMerDistinct = 0.975”. PacBio reads were also corrected using *Canu* (Schmidt et al., 2017) with the same parameters except for “-correctedErrorRate = 0.045”. The corrected Nanopore and PacBio reads, were aligned to the assembly using *NGMLR* version 0.2.7 (Sedlazeck et al., 2018) with the following parameters “-x ont –no-smallinv”.

The GOGG method (He and Bancroft, 2018), which uses the distribution pattern of gene allelic SNPs of 134 lines in a mapping population the AC *Brassica* genomics platform (He et al., 2015) to detect large structural misassemblies, was performed. The results were manually inspected for blocks with deviating patterns. The collinearity of putatively misassembled blocks with the AC pantranscriptome, *Arabidopsis thaliana* and *Thellungiella parvula* were used as supporting evidence for misjoins. Breakpoints of misjoins were resolved by inspecting the alignments of long reads with *IGV* (Robinson et al., 2011) in putatively misassembled regions. When supported by clear alteration of read coverage, the breakpoints were cut to isolate the misassembled blocks using *fastasubseq* under *Exonerate* suite (Slater and Birney, 2005). Gene annotation was updated to the corrected assembly using *flo* (Pracana et al., 2017) which implements the UCSC tool *liftover* (Kuhn et al., 2013).

### Evaluation of Gene Set Completeness

The completeness of genic regions were evaluated with two standard assessment pipelines, *BUSCO* v4.0.4 (Simão et al., 2015) and *PLAZA coreGF* (van Bel et al., 2012), where the presence of highly-conserved orthologs was used to score an assembly. *BUSCO* was performed on the genome assembly using the lineage dataset brassicales_odb10. Results were plotted using the generate_plot.py script of *BUSCO*. Since the coreGF python script does not set alignment threshold, predicted proteins were first aligned to PLAZA_2.5_proteome.fasta using *BLASTP* (Camacho et al., 2009), and only alignment with above 60 percentage identity and “-evalue 10e-5 –qcov_hsp_perc 60” were used to calculate for weighted score against the “greenplants” coreGFs.

Single-ended mRNA sequencing data (SRR3134083) was aligned to the assembled genome using *HISAT2* (Kim et al., 2019) and converted to intron boundaries using *bam2hints* (Augustus 3.2.1) (Hoff and Stanke, 2013, 2018). Positions were compared using *windowBed* (v2.25.0) (Quinlan and Hall, 2010).

Predicted proteins were evaluated with presence of known protein domains using *InterproScan* v5.33-72.0 (Jones et al., 2014) with the following parameters “interproscan.sh -appl TIGRFAM, PANTHER, Pfam, PrositeProfiles, PrositePatterns –iprlookup –goterms –pa”.

The predicted coding sequences were also aligned to the A and C genome-based ordered pan-transcriptome (He et al., 2015) using *BLASTN* (Camacho et al., 2009) with the following parameters “-qcov_hsp_perc 60, -evalue 10e-5” and only alignments coverage of 60% to both target and query, a percentage identity of 60% and above were counted.

### Genome-to-Genome Comparison

Whole genome alignment between assemblies was performed using the graphical interface of *D-Genies* (Cabanettes and Klopp, 2018), which invokes *Minimap2* (Heng Li, 2018) internally and generates dotplots. Dotplots were displayed by applying a match size filtering, where matches that were grouped in the bins of smaller sizes (first and second out of 7 bins) were removed. Genomes used were *B. napus* Darmor-*bzh* v 4.1 (Chalhoub et al., 2014), *B. rapa* and *B. oleracea* (Belser et al., 2018).

Collinearity between genomes was identified by first obtaining orthologous genes and then these genes were used as anchor to detect synteny and collinearity using *MCScanX* (Wang et al., 2012). Since the *MCScanX* recommends around five hits per transcript, the alignment run was performed in two steps: (1) aligning all transcripts of both genomes to themselves and to each other using *BLASTN* (Camacho et al., 2009) with following parameters (2) filtering hits by alignment coverage and percentage identity of 80% and above. *MCScanX* was performed with parameters “match_score 50, match_size 10, gap_penalty -1, overlap_window 10, e_value 1e-05, max_gaps 10”. The output was then plotted for visualization using *SynVisio*^3^ to display blocks with final score of 10000 and above.

## Data Availability Statement

The datasets generated for this study can be found in the NCBI BioProject PRJNA587046 (sequencing reads and optical map) and Zenodo https://doi.org/10.5281/zenodo.3524259 (unpublished genetic maps, assembled genome and annotation results).

## Author Contributions

HL conducted the analysis and drafted the manuscript. HC and CO constructed the optical map and HC sequenced the Nanopore long reads. CO, FD, and AA constructed genetic maps. RS and HL conceived the study. All critically reviewed and edited the manuscript.

## Conflict of Interest

AA and FD were employed by the company NPZ Innovation GmbH.

The remaining authors declare that the research was conducted in the absence of any commercial or financial relationships that could be construed as a potential conflict of interest.

## Footnotes

1 https://github.com/jts/nanopolish

2 https://github.com/PacificBiosciences/GenomicConsensus

3 https://kiranbandi.github.io/synvisio/
