# Supplementary material for: Chromosome-Scale Assembly of Winter Oilseed Rape Brassica napus
Source: Front Plant Sci. 2020 Apr 28;11:496. doi: 10.3389/fpls.2020.00496 (PMC7202327; doi:10.3389/fpls.2020.00496)
Supplement: Supplementary file 1 [file Presentation_1.PPTX]

## Slide 1
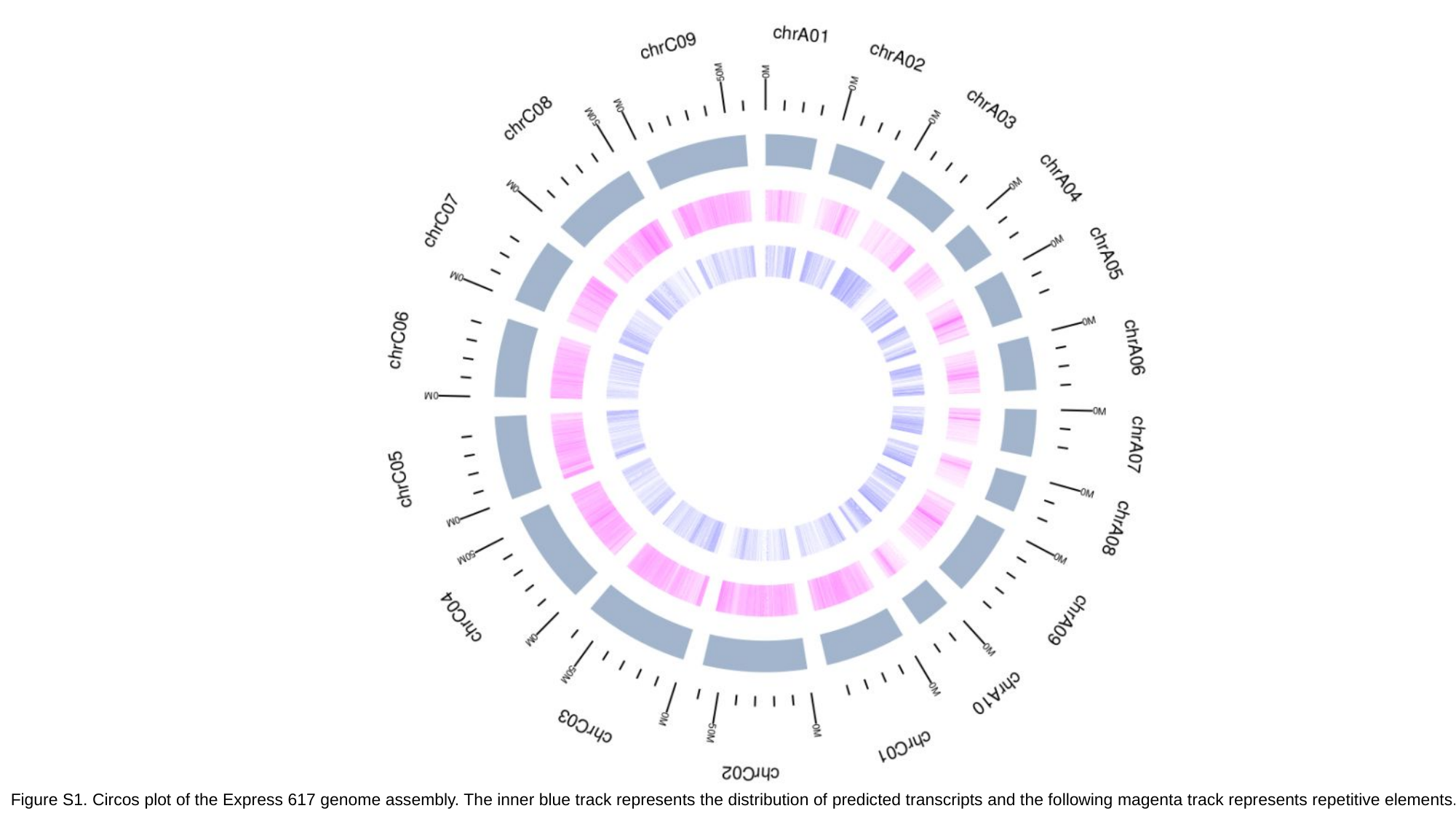

Figure S1. Circos plot of the Express 617 genome assembly. The inner blue track represents the distribution of predicted transcripts and the following magenta track represents repetitive elements.

## Slide 2
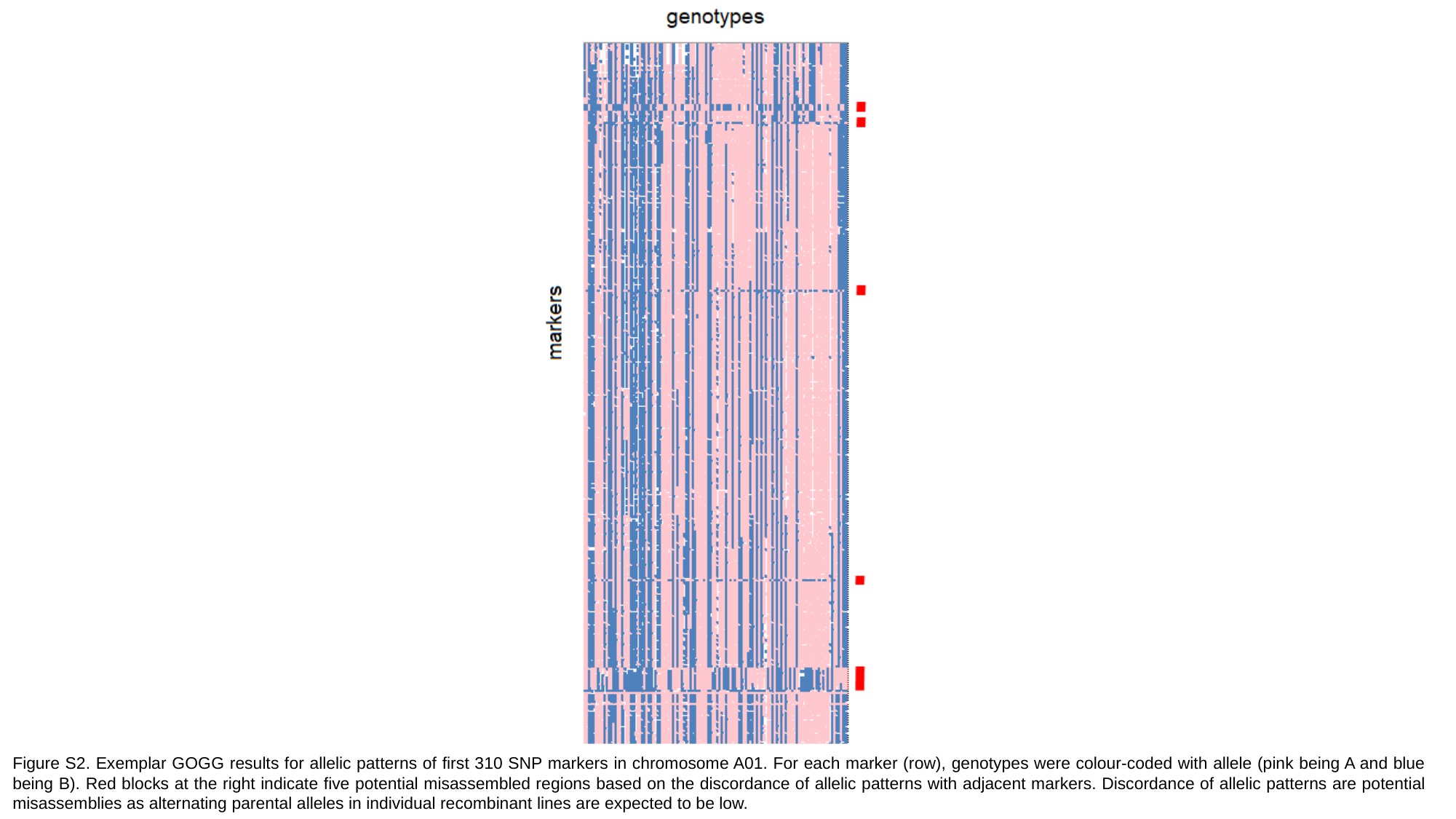

Figure S2. Exemplar GOGG results for allelic patterns of first 310 SNP markers in chromosome A01. For each marker (row), genotypes were colour-coded with allele (pink being A and blue being B). Red blocks at the right indicate five potential misassembled regions based on the discordance of allelic patterns with adjacent markers. Discordance of allelic patterns are potential misassemblies as alternating parental alleles in individual recombinant lines are expected to be low.

## Slide 3
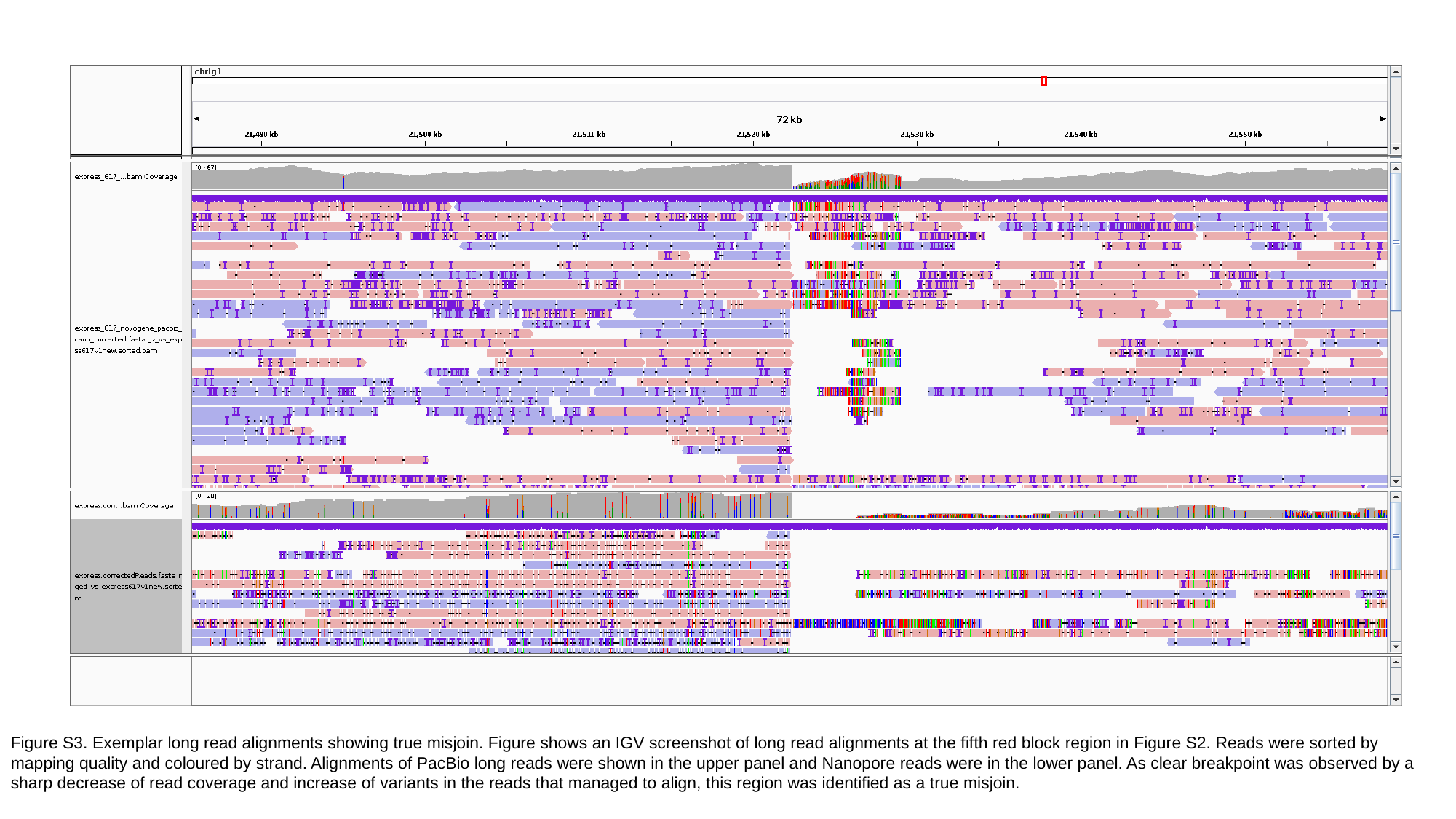

Figure S3. Exemplar long read alignments showing true misjoin. Figure shows an IGV screenshot of long read alignments at the fifth red block region in Figure S2. Reads were sorted by mapping quality and coloured by strand. Alignments of PacBio long reads were shown in the upper panel and Nanopore reads were in the lower panel. As clear breakpoint was observed by a sharp decrease of read coverage and increase of variants in the reads that managed to align, this region was identified as a true misjoin.

## Slide 4
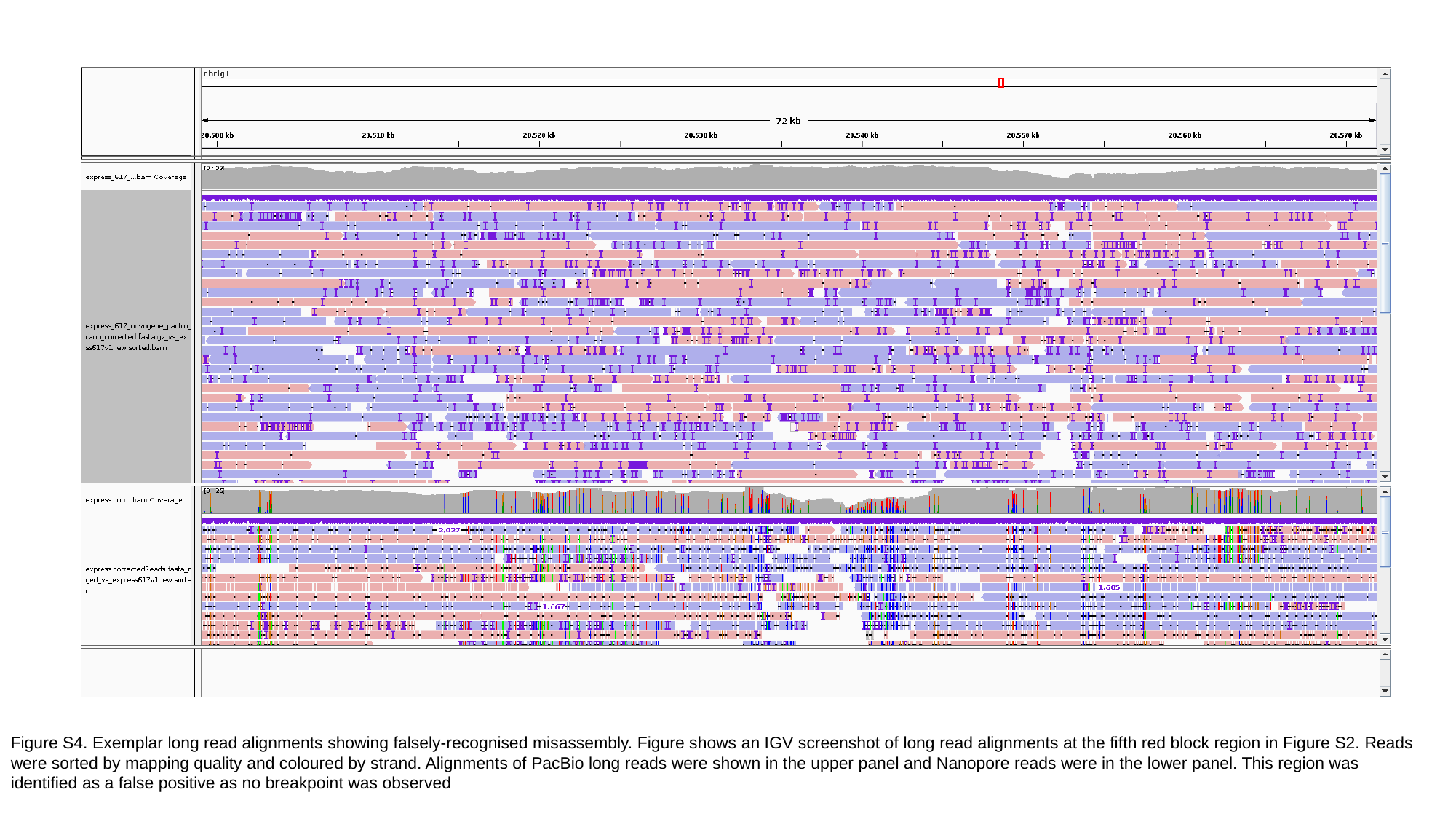

Figure S4. Exemplar long read alignments showing falsely-recognised misassembly. Figure shows an IGV screenshot of long read alignments at the fifth red block region in Figure S2. Reads were sorted by mapping quality and coloured by strand. Alignments of PacBio long reads were shown in the upper panel and Nanopore reads were in the lower panel. This region was identified as a false positive as no breakpoint was observed

## Slide 5
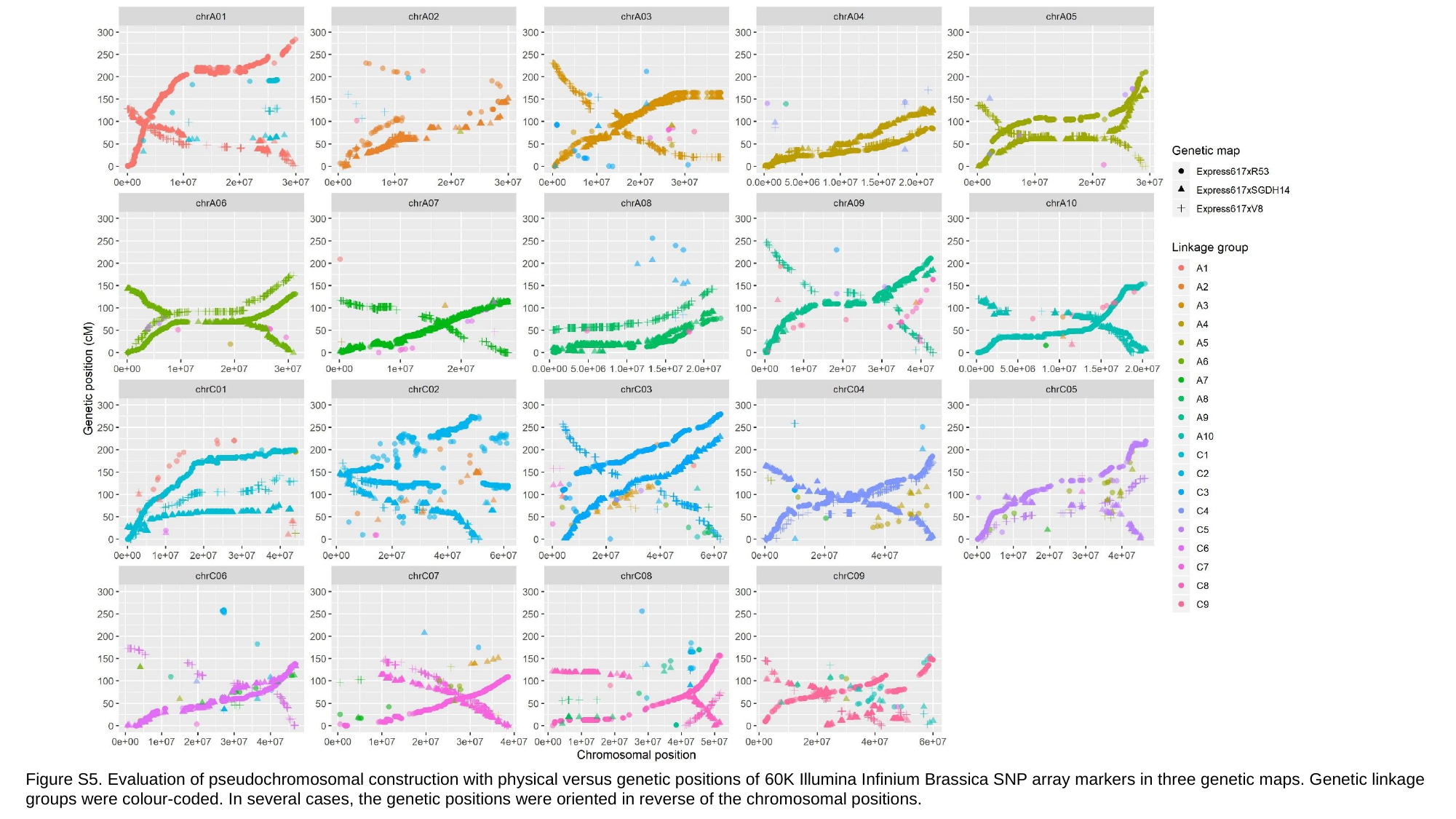

Figure S5. Evaluation of pseudochromosomal construction with physical versus genetic positions of 60K Illumina Infinium Brassica SNP array markers in three genetic maps. Genetic linkage groups were colour-coded. In several cases, the genetic positions were oriented in reverse of the chromosomal positions.

## Slide 6
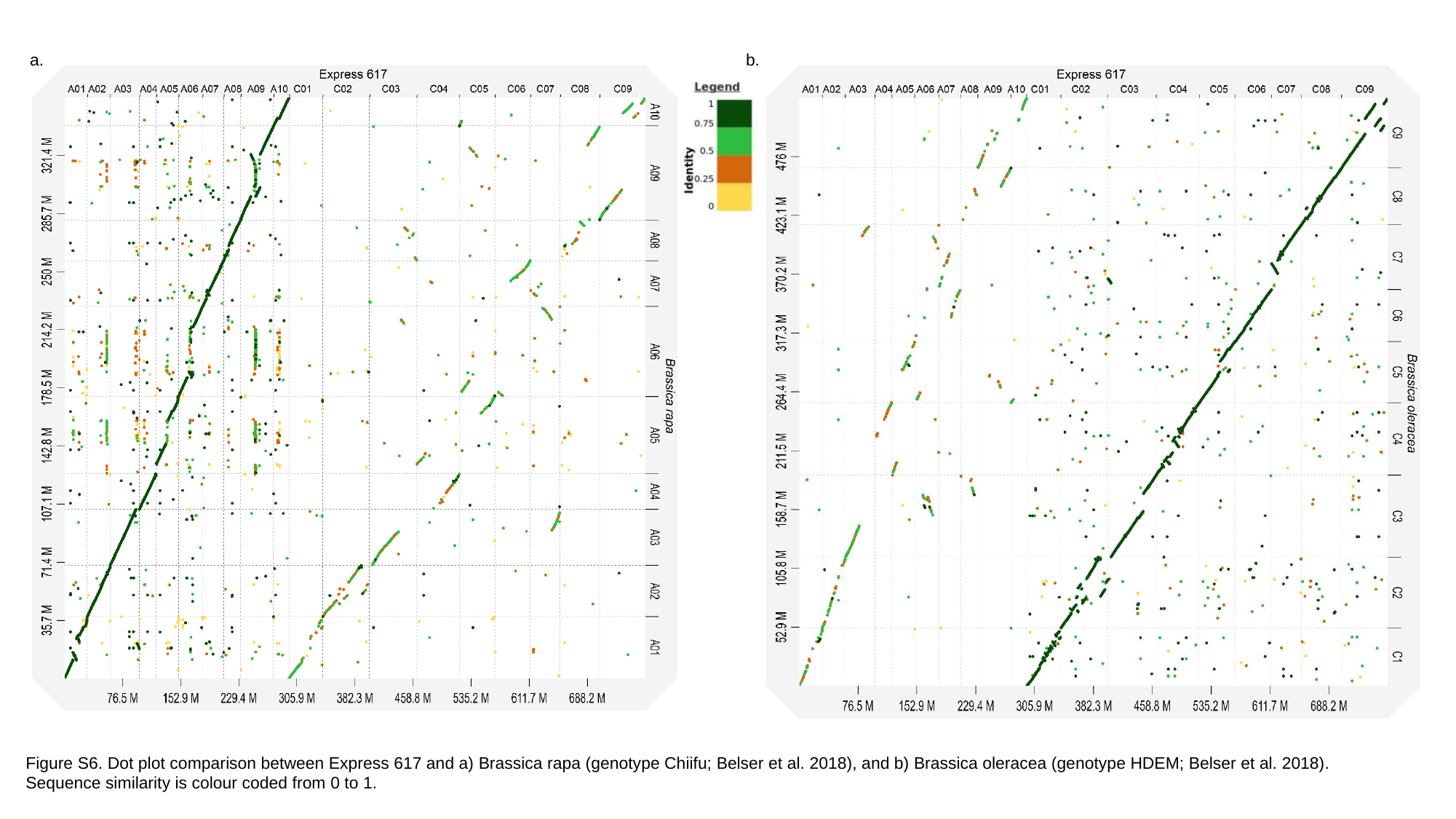

a.
b.
Figure S6. Dot plot comparison between Express 617 and a) Brassica rapa (genotype Chiifu; Belser et al. 2018), and b) Brassica oleracea (genotype HDEM; Belser et al. 2018). Sequence similarity is colour coded from 0 to 1.

## Slide 7
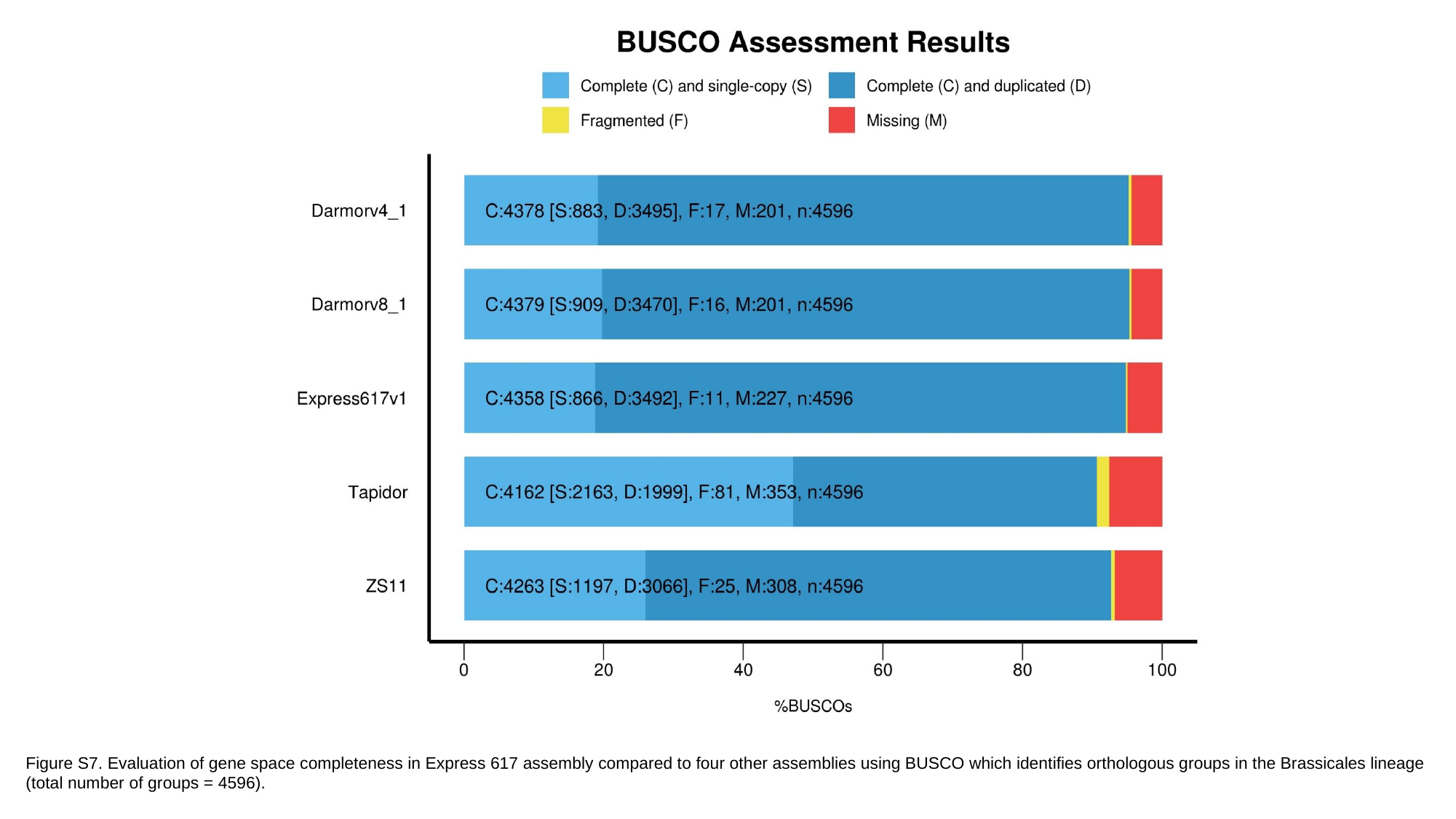

Figure S7. Evaluation of gene space completeness in Express 617 assembly compared to four other assemblies using BUSCO which identifies orthologous groups in the Brassicales lineage (total number of groups = 4596).
