# Supplementary material for: Chromosome-Scale Assembly of Winter Oilseed Rape Brassica napus
Source: Front Plant Sci. 2020 Apr 28;11:496. doi: 10.3389/fpls.2020.00496 (PMC7202327; doi:10.3389/fpls.2020.00496)
Supplement: Supplementary file 2 [file Table_1.DOCX]

Table S1. Whole genome sequencing data used in assembly.

| Platform | Sequenced coverage (estimated genome size used = 1Gb) | Insert size (bp) | SRA ID |
| --- | --- | --- | --- |
| Illumina paired-end  (read length = 150 bp) | 78.5 | 350 | SRR10382360  SRR10382369-SRR10382371 |
|  | 88.0 | 450 | SRR10382363-SRR10382366 |
|  | 48.1 | 2000 | SRR10382372-SRR10382373 |
|  | 51.5 | 5000 | SRR10382367 |
|  | 32.2 | 10000 | SRR10382361-SRR10382362 |
| 10X Genomics | 133.2 | - | SRR10383376-SRR10383381  SRR10383384-SRR10383400 |
| PacBio Sequel | 54.5 | - | SRR10382368 |

Table S2. Statistics of Nanopore reads generated

| Total bases (bp) | 23379820717 |
| --- | --- |
| Number of reads | 1064504 |
| Read length N50 (bp) | 32597 |
| Median read quality | 11.4 |
| Average read quality | 10.7 |

Table S3. Characteristics of placed and unplaced scaffolds in the Express 617 assembled genome

| Type of assembled sequences | Number of scaffolds/ pseudochromosome | Total length | Total length of predicted repetitive elements | Total number of predicted genes | Example of fasta header |
| --- | --- | --- | --- | --- | --- |
| Pseudochromosome | 19 | 764629779 | 299730058  (39.2%) | 84108 | chrA01 |
| Unplaced scaffolds with assigned chromosome | 42 | 37883135 | 11660527  (30.8%) | 2946 | R121_chrC07 |
| Unplaced scaffolds with unknown chromosome | 1370 | 122582145 | 42052124  (34.3%) | 2803 | R105 |
|  |  |  |  |  |  |

Table S4. Classification of repetitive elements found in Express 617 assembly.

| Repeats | Class |  | Number of elements | Total length (Mb) | Percentage out of all masked bases | Percentage out of total genome length |
| --- | --- | --- | --- | --- | --- | --- |
| Transposable elements |  |  |  |  |  |  |
|  | LTR |  |  | 137.0 | 28.3 | 14.8 |
|  |  | Copia | 48107 | 58.4 | 16.8 |  |
|  |  | Gypsy | 64060 | 77.2 | 22.2 |  |
|  |  | Cassandra | 2323 | 0.9 | 0.2 |  |
|  |  | ERV1 | 239 | 0.1 | 0.05 |  |
|  |  | Others | 1962 | 0.4 | 0.1 |  |
|  | Non-LTR subclass I |  |  | 24.7 | 4.9 | 2.7 |
|  |  | LINEs | 34281 | 22.8 | 6.5 |  |
|  |  | SINEs | 10939 | 1.9 | 0.5 |  |
|  | Non-LTR subclass II |  |  | 79.2 | 13.5 | 8.6 |
|  |  | CMC-EnSpm | 22942 | 35.2 | 10.1 |  |
|  |  | MULE-MuDR | 18200 | 11.0 | 3.2 |  |
|  |  | PIF-Harbinger | 15496 | 5.9 | 1.7 |  |
|  |  | TcMar-Stowaway | 14769 | 3.2 | 0.9 |  |
|  |  | TcMar-Tc1 | 93 | 0.0 | 0.009 |  |
|  |  | hAT-Ac | 36995 | 13.4 | 3.9 |  |
|  |  | hAT-Charlie | 2255 | 0.9 | 0.3 |  |
|  |  | hAT-Tag1 | 10054 | 2.2 | 0.6 |  |
|  |  | Helitron | 7913 | 4.4 | 1.3 |  |
|  |  | Others | 9702 | 3.0 | 0.9 |  |
|  | Unclassified |  | 256463 | 88.6 | 25.5 | 9.6 |
| Satellites |  |  | 5735 | 1.6 | 0.5 | 0.2 |
| Simple repeats |  |  | 224248 | 13.4 | 3.9 | 1.5 |
| Low complexity sequences |  |  | 49765 | 2.6 | 0.8 | 0.3 |
| Total masked sequences |  |  |  | 347 | 100 | 37.5 |

Table S5. Alignment results of short Illumina and long Nanopore reads to Express 617 genome

| Libraries | Total read pairs/reads | Number of mapped reads/pairs | Number of mapped read pairs with zero mismatch and gap | Error rate measured by ratio of mismatch to mapped bases (unpaired-map allowed) |
| --- | --- | --- | --- | --- |
| SRR10382360 | 50444379 | 49230444 | 45458404 (90%) | 76157273/14853077610 |
| SRR10382371 | 57949895 | 56562570 | 52237407 (90%) | 90533178/17065712535 |
| SRR10382370 | 54671352 | 53357984 | 49260784 (90%) | 77471338/16096607865 |
| SRR10382369 | 53625980 | 52329813 | 48316074 (90%) | 78206715/15786879650 |
| SRR1030294 | 89492069 | 80569949 | 77619413 (87%) | 38625231/10661279989 |
| Total short reads | 306183675 | 292050760 | 272892082 (89%) | 0.48% |
| Corrected Nanopore reads | 652920 | 562142 (86%) | - | - |
| Corrected PacBio reads | 4348944 | 4328786 (99%) | - | - |
